# Automatic sleep spindles identification and classification with multitapers and convolution

**DOI:** 10.1093/sleep/zsad159

**Published:** 2023-06-09

**Authors:** Ignacio A Zapata, Peng Wen, Evan Jones, Shauna Fjaagesund, Yan Li

**Affiliations:** School of Mathematics, Physics and Computing, University of Southern Queensland, Darling Heights, Australia; School of Engineering, University of Southern Queensland, Toowoomba, Australia; Health Hub Doctors Morayfield, Queensland, 4506, The University of the Sunshine Coast, Queensland, 4556, Australia; Health Developments Corporation, Health Hub Morayfield, Queensland, 4506, University of the Sunshine Coast, Sippy Downs, Queensland, 4556, Australia; School of Mathematics, Physics and Computing, University of Southern Queensland, Darling Heights, Australia

**Keywords:** multitapers, spectral estimation, sleep EEG, sleep spindles, spectra density estimation (SDE)

## Abstract

Sleep spindles are isolated transient surges of oscillatory neural activity present during sleep stages 2 and 3 in the nonrapid eye movement (NREM). They can indicate the mechanisms of memory consolidation and plasticity in the brain. Spindles can be identified across cortical areas and classified as either slow or fast. There are spindle transients across different frequencies and power, yet most of their functions remain a mystery. Using several electroencephalogram (EEG) databases, this study presents a new method, called the “spindles across multiple channels” (SAMC) method, for identifying and categorizing sleep spindles in EEGs during the NREM sleep. The SAMC method uses a multitapers and convolution (MT&C) approach to extract the spectral estimation of different frequencies present in sleep EEGs and graphically identify spindles across multiple channels. The characteristics of spindles, such as duration, power, and event areas, are also extracted by the SAMC method. Comparison with other state-of-the-art spindle identification methods demonstrated the superiority of the proposed method with an agreement rate, average positive predictive value, and sensitivity of over 90% for spindle classification across the three databases used in this paper. The computing cost was found to be, on average, 0.004 seconds per epoch. The proposed method can potentially improve the understanding of the behavior of spindles across the scalp and accurately identify and categories sleep spindles.

## Graphical Abstract

**Graphical Abstract:**
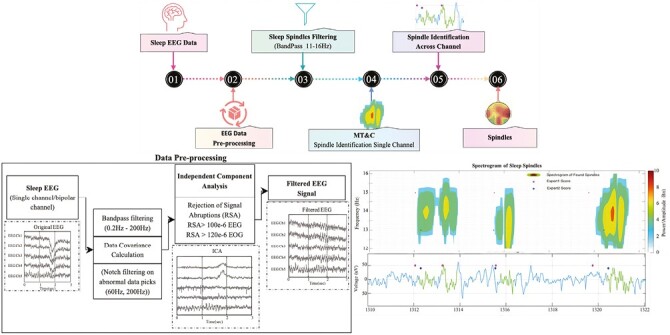

**Statement of Significance**

Sleep spindles are a pattern of brain waves that occur during nonrapid eye movement. They have been presumed to correlate with memory consolidation, sleep quality and aging significantly. To identify and classify spindles, we developed a method named “spindles across multiple channels” (SAMC) that combines multiple channels to identify spindles. This method does not require training or expert’s labels. Instead, it uses the definitions and parameters of the spindles from the Rechtshaffen and Kales sleep criteria. The SAMC method uses time–frequency analysis to generate spindle-like wavelets by using multitapers (MTs) technology. The wavelets from the MTs are then convoluted with the EEG data to extract the spindles components. This process is performed on each EEG channel, and then the spindles are scored if there is a spindle presence agreement across channels. Overall, this method provides a substantial spindle classification improvement over other methods, with easy use for analyzing and tracking spindle behaviors.

## Introduction

Polysomnography (PSG) has been under continuous research for many years. It aims to understand and identify the links between neuronal behaviors with body functions. Sleep spindles are one of those neuronal comportments that have attracted the researcher’s interest because of their connections with nonrapid eye movement (NREM), memory consolidation, and mental and physical problems [1, 2].

Understanding sleep mechanisms and their relationships with human brain activities have progressed over the past decades. Research shows that the structure and patterns of electrophysiological features are associated with certain neurological functions or conditions [1, 2]. Limitations, however, remain regarding the associations of specific neuronal behaviors with brain functions. One of those limitations is how to interpret and analyze brain wave structures, like spindles [3, 4].

This paper analyses sleep spindles and their identification from sleep electroencephalogram (EEG) data. The proposed method is a time–frequency analysis using a multitapers and convolution (MT&C) method [5–7] to calculate the spectral estimation of spindles’ characteristics in EEG data. Sleep spindles are bursts of energy ranging between 9 and 16 hertz (Hz) frequencies with a pyramidal-like structure that wanes and waxes its oscillations between 0.5 and 2 seconds (s). Spindles are characterized as thalamocortical circuits because they are generated in the thalamus and move forward to the brain’s cortex. The shape and duration of sleep spindles are based on the reciprocal interactions between the cortex and the thalamus [8–13].

Up to now, no explicit brain functions are associated with sleep spindles. However, spindles are widely assumed to be associated with memory consolidations and plasticity [14]. Another uncertainty surrounding sleep spindles is their frequency range. Existing studies have defined spindle transient waveforms differently, with some defining them in frequencies between 9 Hz and 16 Hz [15], while others conceptualized spindles in the frequency range of 11 Hz and 15 Hz [8, 16, 17]. Most studies agreed that there are two types of spindles: slow spindles ranging between 9 Hz and 11 Hz or 13 Hz, and fast spindles ranging between 13 and 15 Hz or 16 Hz [18–24].

This research uses the frequency range of 11–16 Hz under the spindle definition and parameters from the Rechtshaffen and Kales sleep criteria (R&K rules) [25], as shown in Table 1. This paper defines a spindle based on the spindle definition and parameters shown in Table 1, which are used to create the tapers for the MT&C method. The tapers are the combination of a kernel function with the parameters of the spindles. Therefore, in this study, the terms of *taper*, *kernel*, and *wavelet* could refer to the same use.

**Table 1.** Spindles parameters used in the proposed method.

Spindles parameters		
Frequency	Time duration	Min amplitude
11–16 Hz	0.5–2.5 s	13 uV

This research aims to develop a new method to interpret, identify, classify, and visualize sleep spindles across multiple EEG channels from subjects, including those with different neurological conditions (unhealthy subjects). Spindles are identified using a method named “spindles across multiple channels” (SAMC), which scores spindles when they are identified across several channels. There are two main reasons for using multichannel EEGs for spindles identification. First, those bio-signals can track the behaviors of spindles across the scalp. Second, it can provide a higher level of certainty as we can isolate spindles from other brain activities with similar wave patterns and structures.

This paper is organized as follows. Section 2 briefs relevant research on sleep spindles and multitapers-based studies. Section 3 introduces the EEG databases (DBs) used in this research. Section 4 describes the MT&C method for identifying sleep spindles. Section 5 presents the research findings from the different experiments. Finally, Section 6 summarizes the research and future work.

## Related Work

Many studies are dedicated to sleep spindles analysis using different methods to identify their characteristics and connections with human physiology. Yet there are still many concerns surrounding sleep spindles, such as:

- The link between spindle types with specific body functions.
- The consistency of identification across subjects with altered neurological conditions,
- The parameters, particularly the frequency range, used to identify sleep spindles.

### Spindles Related Applications

One of the most prominent roles of sleep spindles is its relationship with the NREM sleep [10, 26–28]. For instance, sleep spindles and k-complexes (KCs) are the hallmarks used to distinguish sleep stage 2 or light sleep from other stages [29, 30]. As reported by [26], sleep spindles and KCs are correlated during stage 2 sleep with an incidence of around 68%. However, the occurrences of KCs and spindles have no associations with any of their physical characteristics or the probability of spindles’ appearance.

For memory consolidation, spindles are believed to play an important role. A study from [28] suggested that for healthy individuals between middle age and older, the spindle density can be used as a marker to establish the stability of the neurophysiological characteristics that play a role in cognitive functions and plasticity. They also implied that the duration of the REM stage is directly associated with the integration of neurotransmitters and neuromodulators, which are fundamental parts of our autonomic nervous system.

In terms of the topography of spindles, an early study by [3] indicated that spindle events were independent and located in different cortical areas. However, recent reports suggested that spindles were identified across different cortical areas, and most events occurred simultaneously [31, 32]. Even though some reports try to explain the topography of spindles, some suggestions indicate that spindles are not coherent in their occurrence as they are not regularly phase-locked or have the same frequency ranges [8, 17]. It has been demonstrated that there are two types of spindles so far. Spindles with low peak frequency in the frontal cortex with anteroposterior gradients in their frequency oscillation range of 9–12Hz are called slow spindles. Spindles with a higher peak frequency with nonphase-locked and between 13 Hz and 15 Hz are known as fast spindles [15, 23, 32, 33].

Sleep spindles are a constant indication of abnormal neuronal brain behaviors, not because of specific elements hidden in the spindle waves but due to their absence in sleep EEG recordings. A study by [34] found that spindles were not as frequent in subjects with Asperger’s syndrome (AS) compared to normal subjects, although all the other elements in sleep data in AS subjects were normal. Similarly, a report by [35] indicated that the sleep data from subjects with autism spectrum disorders (ASD) were like normal subjects, except for sleep spindles, which were notoriously less in ASD subjects [36]. Another study by [37] showed that subjects with mental retardation have notorious abnormalities across all sleep stages compared to healthy subjects, especially in spindles and KCs, which have disreputable atypical events and low-rate patterns.

### Multitapers Related Applications

Multitapers (MTs) are mechanisms of exploration that use time–frequency analysis to extract detailed information from signals and map specific elements of an object or concern. As implemented in [5], MTs were used to identify different frequencies, power, and time of an event present in sleep EEG data to generate features that were directly associated with the sleep physiology based on the R&K rules [25]. They classified sleep stages with an average rate of 87% with the option of visualizing them using a spectral estimation from each epoch. It was seen that MTs were able to represent specific events (e.g., frequency and power).

Babadi and Brown [38] presented a detailed analysis of a spectral and a standard nonparametric spectral estimation from MTs. They applied an MTs-based method to analyze anesthetic and sleep EEG data. They showed that by specifying the spectral resolution of the tapers, the frequencies outside of the taper range resolution became blurry, allowing them to identify only elements within the spectral resolution of the taper. That study gave an insight into how MTs-based methods could identify an accurate spectral estimation for different types of EEG signals [39].

A neurophysiology review from sleep EEG data was presented by [4] using a spectral analysis generated by MTs spectrograms. They demonstrated how an MTs-based method could be used as an effective tool to present a more defined way to visualize EEG data for producing better and faster results in classifying sleep stages. They found that the spectrograms allowed them to identify the underlying oscillatory mechanisms in each sleep stage, creating a visual representation that was easier to map with their hypnogram corresponding to the original signal. Their results showed a very close relationship between expert labels and the spectrograms produced by the MTs method.

### Existing Studies for Result Comparisons

The performances of the proposed method in this paper are compared with other studies that used similar methods for spindles identification. The article by Wamsley [16] and implemented in [15] used a wavelet-based algorithm to detect spindles automatically. The algorithm was based on a spectral estimation from a fast Fourier transform, applying a Hanning window to three-second epochs. In the case of [15], they did not compare their results with other studies. The proposed method in this paper is applied to the databases provided by the authors and compared to their results [15].

## Experimental Data

This study uses three open-access databases (DBs) to identify spindles by applying the proposed method. All three DBs include spindle labels from experts, as seen in Table 2. The first open-access DB is the NAP EEG BD from Open Science Framework (OSF), published in [12]. The second open-access DB is the Dreams DB from ZENODO, published in [40, 41]. The third open-access DB is the Montreal Archive of Sleep Studies (SS2-MASS), published in [42].

**Table 2.** Spindles available on each database and the number of subjects

Number of spindles in the databases		
Database	Number of subjects	Spindles
NAP	22	2528
SS2-MASS	19	22254
Dreams	8	475

### NAP EEG DB

The NAP EEG DB contains the EEG recordings from 22 subjects between 18 and 43 years old, who completed memory tasks before their naps. Each recording includes 62-channel data and two electrooculograms (EOG) electrodes with a sample rate of 1000 Hz. The data from each subject were collected on two separate days. The annotations on the DB are as awake, stage 1, stage 2, stage 3, spindles and KCs.

All annotations were based on 30-second epochs and manually scored. Spindles were scored by visual inspection of anterior–posterior brain regions using the data from channels of F3, F4, C3, C4, O1, and O2, which were positioned and recorded following the 10–20 EEG international system [12].

### SS2-MASS C1 DB

The SS2-MASS DB from the MASS-C1 DB was published in 2014. It contains 19 subjects’ polysomnographic recordings from three different laboratories of the Centre for Advanced Research in Sleep Medicine, Montreal, Canada. The subjects are between the ages of 18 and 33. The data were recorded using Harmonie software, with an amplifier system Grass Models 12 and 15. This research uses the SS2 DB as it is the only one that contains spindles’ labels. The EEG data from the SS2 DB has 19-channel montage (C3, C4, Cz, F3, F4, F7, F8, O1, O2, P3, P4, Pz, T3, T4, T5, T6, Fp1, Fp2, Fpz). It also contains four EOGs, one EMG, one ECG and one Respiratory thermistance. The sample rate of the SS2 DB is 256 Hz for all channels, except for the respiratory thermistance, which was recorded at 54 Hz.

The hypnograms from the SS2 DB contain the labels for spindles and KCs from two experts who manually labeled them using the R&K rules [25]. The labels of the spindles and KCs include approximated coordinates of the start and end of the events [42].

### Dreams DB

The Dreams DB contains 30 minutes of sleep recordings from EEG, EOG, and EMG channels for eight subjects between the ages of 31 and 53. The data from that DB has not been filtered. The subjects present different pathologies like dyssomnia, restless legs syndrome, insomnia, and apnoea/hypopnoea syndrome. The DB contains three EEG channels (Cz-A1 or C3-A1, Fp1-A1, and O1-A1), two EOGs (P8-A1 and P18-A1) and one submental EMG. The sample rates are 200 Hz, 100 Hz and 50 Hz, respectively. The data were scored for sleep stages using the R&K rules [25, 40].

## Methodology

This research implements the SAMC method to identify and classify spindles. The SAMC method uses the MT&C method [5] to extract the key spindles information from different channels from the three sleep EEG DBs. The proposed method identifies the signal power on frequencies between 11 and 16 Hz. The spindle-like waves are then analyzed and classified in terms of their duration. Identified spindle waves across multiple channels are transformed into logical data (zeros for nonspindle waves and ones for spindle-like waves) to map the spindle-like waves’ agreement and duration across all the EEG channels. Independent epochs are rated as a spindle only if they are consistently identified across a particular number of channels surpassing the minimum power and duration criteria of spindles.

### Data Preprocessing

**Figure 1.**
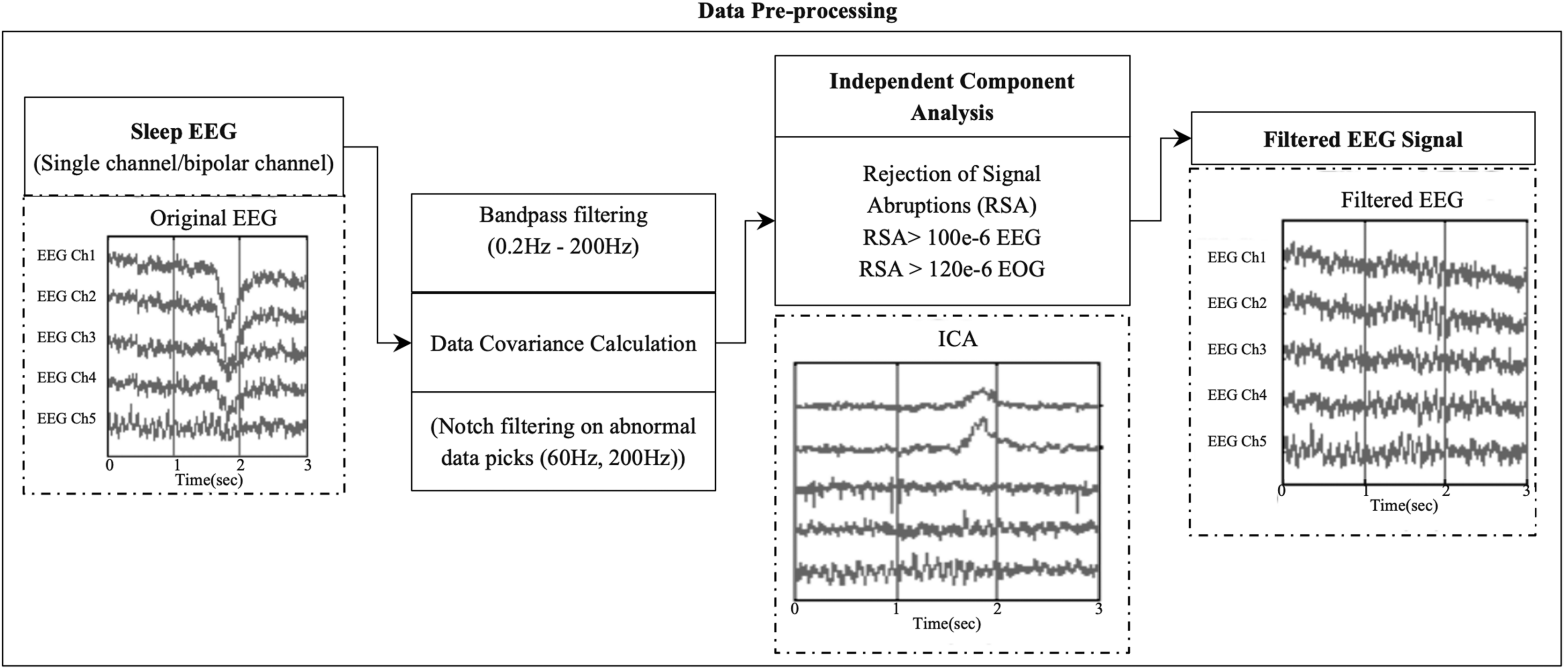
Data pre-processing.

**Figure 2.**
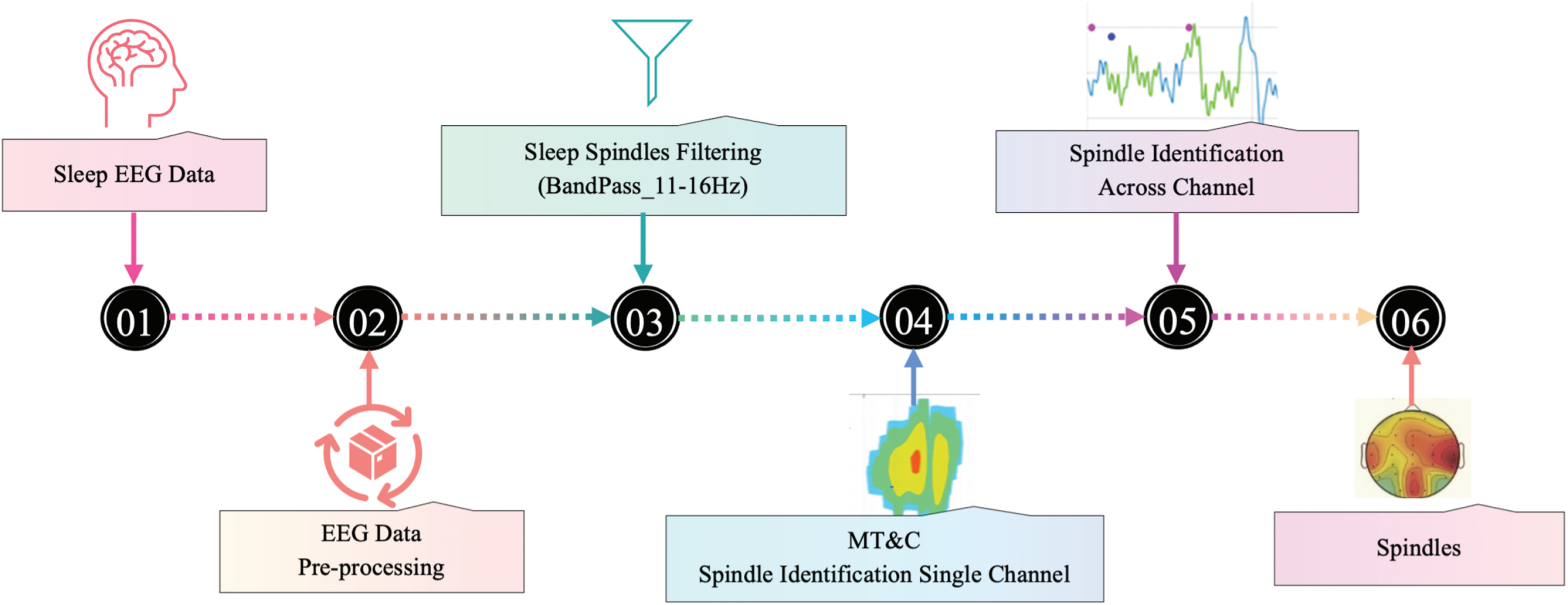
Sleep spindles classification algorithm: (1) Sleep EEG data from databases. (2) Sleep EEG data preprocessing using muscle movement detections from MNE Python Library. (3) Data filtering based on spindles frequency parameters using a Bandpass filter (11–16 Hz). (4) Spindle Identification on single-channel based on parameters from Table 1. (5) Spindle Identification Across Channels (SAMC method) based on a rule of a spindle wave from one channel has 25% agreement with another channel. (6) Resulting spindles are generated in terms of Power, Frequency range, and Time-duration.

All EEG data are preprocessed using the MNE Python Library [43]. As shown in Figures 1 and 2, the EEG data are filtered using a bandpass between 0.2 Hz and 200 Hz. Then the peaks of every channel are computed to generate the covariance. Simultaneously, a notch filter is applied based on the peaks found in the data. After that, chunks of data defined as muscle movements are removed. Removing epochs with muscle movements is based on abnormal amplitude or frequency peaks (characterized in the awake stage) across channels using an independent component analysis (ICA) estimation.

The ICA algorithm separates the EEG signals into statistically independent components. The components in the ICA algorithm are individual signals that were combined during their recording [44–46].

### The MT&C Method for Identifying Spindle Waves

The MT&C method is implemented to calculate the sleep EEG spectral density estimation (SDE) using tapers that simulate the characteristics of fast and slow spindles (refer to the spindle parameters in Table 1). The SDE accentuates the signal time–frequency characteristics based on the parameters of the tapers. The tapers are wavelets generated using a Gabor kernel, which is convoluted with the signal, intending to highlight the spectra density of the spindles in sleep EEG data [5]. The tapers in the MT&C are generated using the Gabor function (1) and the parameters of the spindles [44–46].


$$\begin{array}{l} g_{k}\left(t\right)=e^{\left(\frac{\left(-t^{2}\right)}{\left(2S^{2}\right)}\right)}\;e^{\left(i\;2\pi \;f_{k}\;t\right)} \end{array}$$ (1)

where $g_{k}\left(t\right)$ is the Gabor taper, which is based on a Gaussian window $e^{\left(\frac{\left(-t^{2}\right)}{\left(2S^{2}\right)}\right)}$ and an imaginary cosine wave $\;e^{\left(i\;2\pi \;f_{k}\;t\right)}$. Here “*t”* in the cosine wave refers to the time duration of the signal, which is the maximum duration of a spindle (2 s), and *f*_*k*_ is the frequency of the taper, which also refers to a Gabor taper [5, 47–49].

Considering that the oscillation of a cosine wave is constant automatic, and infinite, it is combined with a Gaussian function to simulate specific characteristics found in the fluctuated signals. The Gaussian function $e^{\left(\frac{\left(-t^{2}\right)}{\left(2S^{2}\right)}\right)}$ behaves like a filter, which only allows passing the oscillations within a section frame. The size of that frame is ruled by an adjustable standard deviation (S) in (2) [5, 48, 49].


$$\begin{array}{l} S=\frac{n}{\left(2\pi \;f_{k}\right)} \end{array}$$ (2)

where *n* is a logarithmical space vector between the logarithm 10^th^ of the maximum number of cycles and the logarithm 10^th^ of the minimum number of cycles. $\;f_{k}$ is the frequency of the signal at level *k*. The number of tapers involved in the MT&C method is based on the number of frequencies evaluated for the spindles [5].

The SDE is computed for each wavelet “*g*,” “*k*” (“*t*”), using a sliding window across the entire sleep EEG signal as expressed in (3).


$$\begin{array}{l} SDE\left(f_{k}\right)=\underset{t=R\left(-\frac{1}{2}R\right)}{\sum }\;\left(g_{k}\left(t\right)\;X_{w}\right) \end{array}$$ (3)

where *t* is in 2-second intervals, and *R* is the sampling rate. $g_{k}\left(t\right)$ is the taper *k*, which is also a kernel function, and $X_{w}$ is the whole EEG signal.

When the kernel function $g_{k}\left(t\right)$ is convoluted with the original EEG signal, it creates a dot-product for each data point in the EEG signal intending to extract the power present in the signal in terms of the taper parameters [5]. The spectral estimation (ES) from the EEG signal provided by the MT&C method contains the information of the spindles regarding frequency, power, and wavelet duration.

### Spindle Identification in One Channel

The first step to identify spindles from the ES data is to identify their amplitude and extract their normalized power, as shown in (4).


$$\begin{array}{l} m=abs\left(SDE\left(f_{k}\right)\right); \end{array}$$

$$\begin{array}{l} p=sqrt\left(m\right); \end{array}$$

$$\begin{array}{l} norm\_power\;=\;\left(\frac{p\;\;\;-\wedge \left(p\right)}{\vee \left(p\right)-\wedge \left(p\right)}\right)\;\;\; \end{array}$$ (4)

where *p* is the power, *m* is the amplitude, and $SDE\left(f_{k}\right)\;\;\;$ is the spectral estimation of the signal. *sqrt(m)* is the square root of the amplitude, ∧(p)is the minimum power and ∨(p)is the maximum power.

Considering that the MT&C method extracts the powers of EEG signals that match the characteristic of its tapers, all the amplitudes that surpass the power of the peaks (threshold of 0.5 in (4)) are selected as potential spindles. Those spindle candidates are then evaluated based on their duration, determined from the starting point of the power peaks that surpass the threshold to the last point of the power peak. Then based on the number of data points present in the power peak wave, if they are between 0.4 second (a 0.1 second tolerance factor is introduced) and 2 seconds, they are classified as spindles in a single channel.

**Figure 3.**
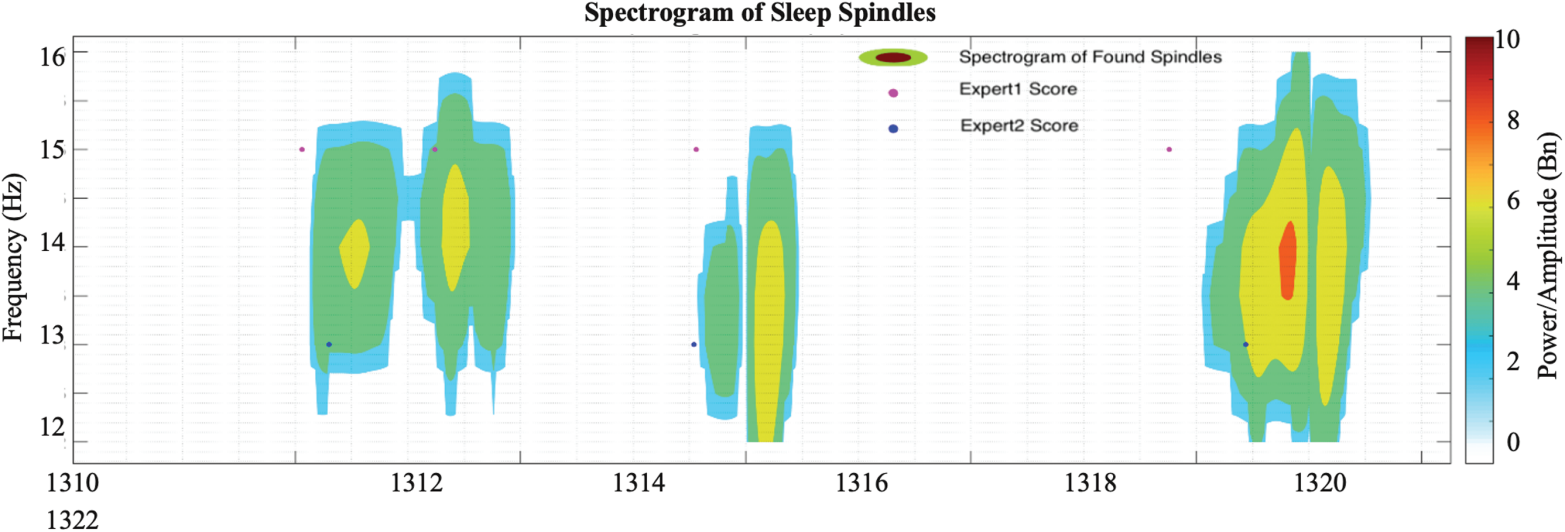
MT&C spectrogram vs expert’s score.

**Figure 4.**
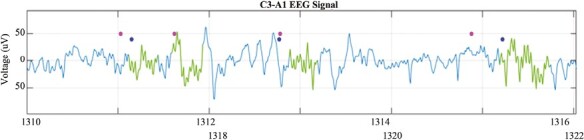
Visualization of classified spindles (green waves) compared to original data (signal in blue) and expert labels (magenta and blue dots).

All the spindles found are gathered in order of events in a set of continuous EEG data. As seen in Figure 3, the spectrogram shows the duration of a spindle, amplitude, and frequency. Fragmented spindles, like the one in the second 1313^th^ in Figure 4, with gaps of 0.10 seconds or less, are scored as a single spindle.

### Spindles Identification Across Channels

After identifying spindles on each channel using the MT&C method, the spindle events are presented in Boolean values (ones and zeros), where zeros represent all the null values (power zero or non-spindle), and ones denote the other values (power of spindle different to zero).

To score the spindles using the SAMC method, at least two channels must agree with each other for at least 25% of the spindle event. The criteria for spindle identification across multiple channels are flexible regarding the agreement percentage between spindles and the number of channels that need to be included.

It is essential to mention that some channels are more sensitive to a specific type of spindles (anterior, posterior, or global spindles). Sometimes, mixing opposite channels could result in false negative identification of spindles across channels. The SAMC can be applied to identify and map the behaviors of spindles across the scalp. It can also classify spindles like anterior, posterior, and global spindles.

### Performance Measurement Metrics

The performance of this study is evaluated using five measurement metrics: Lin’s Concordance Correlation Coefficient (CCC), agreement rate (AR), positive predicted value (PPV), F-measure and sensitivity. Specificity and accuracy are not evaluated for spindle identification because experts usually mark the spindle events that match its definitions/characteristics on the bio-signals like EEGs (True Positives). Most bio-signals are nonspindle events. That means that it is insignificant to identify non-spindles separately. Therefore, true negatives (TN) are not determined. Consequently, specificity and accuracy are not evaluated in this research [50].

In this paper, the CCC method compares the agreement between two entities of the same variable to show the concordance between the results by the proposed method and the scores from the experts. The CCC and AR are also used in this study to analyze the agreement between experts’ scores.

Assuming that from *n* observations, a bivariate set from the same variable is selected *(subscripts x and y)*, with a correlation *ρ*, variances *φ*_*x*_^*2*^ and *φ*_*y*_^*2*^ and the means of *μ*_*x*_ and *μ*_*y*_ [51–55]. Here, *X* and *Y* represent the number of spindles identified in a dataset by two different scorers or methods (expert1 and expert2 or by an expert and the proposed method). The CCC between two entities is defined as:


$$\begin{array}{l} \rho_{c}=\frac{2\rho \;\phi_{x}\;\phi_{y}}{(\mu_{x}-\mu_{y})+\;\phi_{x}^{2}+\phi_{y}^{2}} \end{array}$$ (5)

where *ρ* is the correlation between variables $\;\phi_{x}$and $\;\phi_{y}$ (the number of spindles scored by the entity *x* and the entity *y* on the same DB).

The AR of the spindles considers only the spindles found across channels that agreed with the expert’s scores. The spindles also must be within the range of ether-identified spindles with an overlap of at least 0.25 s. It means that the spindles must overlap 0.25 s to score it as a spindle. The AR is defined as:


$$\begin{array}{l} AR(\%)=\frac{n_{1}+n_{n}\;}{N_{1}+N_{n}}*100 \end{array}$$ (6)

where $N_{1}+N_{n}$ is the number of spindles that match both experts, and $n_{1}+n_{n}$ is the number of spindles found by the proposed method that match the spindles identified by the experts [52, 56].

Sensitivity is to evaluate the correctness of measurement in terms of true positives (TP) and false negatives (FN), as shown in Eq. (7). The TPs for this study are conceptualized as the spindles identified by the proposed method that match the expert spindles for at least 0.25 seconds. The FNs are the spindles scored by an expert but not found by the proposed method [51, 57, 58].


$$\begin{array}{l} Sensitivity\;\;\;=\frac{TP}{TP+FN}\;\;\;\;\;\;\;\;\;\;\;\;\;\;\; \end{array}$$ (7)

The PPV shows the probability that the existence of a condition is present on a subject. Apart from the TP, the PPV also uses false positives (FPs) to find its value, as shown in Eq. (8) [50, 57]. An FP refers to the spindles found by the method but not by an expert.


$$\begin{array}{l} PPV\;\;\;=\frac{TP}{TP+FP}\;\;\;\;\;\;\;\;\;\;\;\;\;\;\;\;\;\;\; \end{array}$$ (8)

The F-measure, which evaluates the binary classification of a system by combining the Precision (PPV) and Recall (Sensitivity) of a model, is defined by Eq. (9) [59].


$$\begin{array}{l} F\;\;\;=\frac{2TP}{2TP+FN+FP}\;\;\;\;\;\;\;\;\;\;\;\;\; \end{array}$$ (9)

## Experimental Results

The spindles detection on a single channel relies primarily on the parameter measurements detected in the data obtained by the MT&C method. When the data are contaminated by any noise like muscle movement, AC power, electronic equipment, a wrong connection of electrodes and even abnormal events like interictal epileptiform spikes, it could trigger an increase of power, causing spindle-like amplitudes [60]. Therefore, EEG data must be adequately denoized and filtered to identify spindles more accurately [61].

The ICA analysis from the MNE Python Library [43], which can remove noisy sections from data, is applied to decrease the number of false positives (FPs) and their impact [44–46].

### Evaluation Criteria

The performance evaluation of the proposed method for identifying spindles is based on the spindle labels marked by any of the experts in the DBs (Expert1 or Expert2), which means that the scores from the experts are combined to compare them with the spindles identified by the proposed method.

Spindle durations are considered for the Dreams and SS2-MASS DBs. In the NAP DB, most durations were not included, or it was set by default to one second [8, 20, 25]. In that case, for the evaluation of spindle identification, each duration of the spindles is set to 1.6 seconds (0.4 seconds before the spindle starts and 1 second after its start).

### Experimental Results

The proposed method is evaluated using data from the SS2-MASS, NAP and Dreams DBs. The experimental results are presented in Tables 3–7 and discussed in the following three sections. The SAMC classification method is evaluated based on the results obtained from the classification of spindles and compared with the labels generated by the experts on each of the DBs.

**Table 3.** Performance Results of Spindles Classification by The Proposed Method (SAMC) With Expert

Performance Results Of Spindle Classification By The Proposed Method (SAMC) With Expert Scores:								
Database	SAMC	Ex1∪Ex2	Agreement	CCC	AR	Sensitivity	PPV	*F*-score
NAP DB	4398	3516	3208	0.72	91%	0.91	0.82	0.86
SS2-MASS DB	18352	15830	15325	0.75	96%	0.96	0.92	0.94
Dreams DB	622	548	538	0.82	98%	0.98	0.91	0.94

**Table 4:** Confusion matrix for the NAP DB

Confusion Matrix for the Nap DB			
		SAMC Classification	
		spindles	Non-spindles
Expert Scores	Spindles	3208 (TP)	308 (FN)
	Nonspindles	665 (FP)	--- (TN)

**Table 5.** Confusion matrix for the SS2-MASS DB

Confusion Matrix for the SS2-MASS DB			
		SAMC Classification	
		spindles	Non-spindles
Expert Scores	Spindles	15325 (TP)	505 (FN)
	Nonspindles	1191 (FP)	--- (TN)

**Table 6.** Confusion matrix for the dreams DB

Confusion Matrix for the Dreams DB			
		SAMC Classification	
		spindles	Non-spindles
Expert Scores	Spindles	538 (TP)	10 (FN)
	Non-spindles	51 (FP)	--- (TN)

**Table 7.** Performance comparison between the SAMC method & other methods

Performance Comparison between SAMC Method & Others				
DB	Method	PPV	F-score	Sensitivity
SS2-MASS DB	Kinoshita *et al* [62].	0.61	0.7	0.77
	Patti *et al* [21].	---	0.69	0.74
	Tsanas *et al* [63].	0.16	---	0.83
	**SAMC**	**0.92**	**0.94**	**0.96**
Dreams DB	Tsanas *et al* [63].	0.33	---	0.76
	Devuyst *et al* [41].	0.74	0.72	0.70
	Kinoshita *et al* [62].	0.55	0.64	0.72
	**SAMC**	**0.91**	**0.94**	**0.98**

#### The Results for the NAP DB

Tables 3 and 4 present the spindle classification results for the NAP DB. The proposed SAMC approach obtained an agreement rate of 91% with impressive results on sensitivity, PPV and *F*-score of 0.91, 0.82, and 0.86, respectively. However, some disparities were detected between the number of spindles identified by the SAMC method and those labelled by the experts, indicating that the SAMC method identified a substantial 20% additional spindles in the NAP DB. Furthermore, our study revealed that approximately 525 spindles annotated by the experts were outside of the defined spindle duration, for example, less than 0.5 seconds in duration or fragmented with a period lower than 0.5 seconds or over 0.25 seconds between fragments, according to the SAMC method,

#### The Results for the SS2-MASS DB

In the case of the SS2-MASS DB, our SAMC classification method was implemented using two EEG channels, namely Cz-A1 and C3-A1.

The results presented in Tables 3 and 5 indicate that the method achieved a spindle classification disagreement of less than 4%. In comparison to other existing studies, our proposed method outperformed the Kinoshita method [62], which utilized a synchro-squeezed wavelet transform for feature extraction and RUS-Boost for classification. The Kinoshita method achieved an average PPV, F-score, and sensitivity of 0.61, 0.7, and 0.77, respectively. Similarly, the Patti method [21], which used a weighted system based on channel combination for feature extraction and clustering of Gaussian mixtures for classification, achieved an average F-score and sensitivity of 0.69 and 0.74, respectively. Lastly, the method proposed by Tsanas [63] used a continuous wavelet transform for feature extraction and classification based on the defined parameters of spindles, achieving an average PPV and sensitivity of 0.16 and 0.83, respectively. Notably, our experimental results demonstrate significant performance improvements in terms of all performance evaluation metrics compared to other studies, as summarized in Table 7.

#### The Results for the Dreams DB

Upon analyzing the Dreams DB, our proposed SAMC method exhibited the highest level of agreement with the combined labels from the two experts. As presented in Tables 3 and 6, the method achieved an average agreement rate of 98%, with a sensitivity, PPV, and F-scores of 0.98, 0.91, and 0.94, respectively. To further evaluate the performance of the SAMC method, its results were compared with other existing methods applied to the Dreams DB. The Tsanas method [63] achieved an average PPV of 0.33 and a sensitivity of 0.76. In contrast, the Devuyst method [40, 41], which used a systematic assessment approach, obtained an average PPV of 0.74 and a sensitivity and an F-score of 0.70 and 0.72, respectively [41]. The Kinoshita method [62], which utilized a synchro-squeezed wavelet transform for spindle classification, achieved an average sensitivity of 0.72, with a PPV and F-score of 0.55 and 0.64, respectively. It is worth noting that the proposed method demonstrated a significant improvement over these existing methods, as illustrated in Table 7.

## Discussion

This study presents a new method for spindle detection in sleep EEG signals. The proposed method combines spectral analysis and machine learning techniques to identify spindles across the scalp using sleep EEG data. The method was tested on three different databases (the NAP, SS2-MASS and Dreams DBs), and its performances were compared with other existing methods in the literature.

Overall, the results show that the SAMC outperforms the existing methods in terms of sensitivity, positive predictive value (PPV) and F-score. The SAMC method was also found to be more robust to inter-expert variability, which is an essential consideration in practical applications.

One of the limitations of this study is that the method was primarily tested on EEG signals from healthy subjects (MASS-DB and NAP-DB) as the data available for subjects with sleep pathologies that contain spindle labels were too limited (Dreams DB: 8 subjects). Meaning that it will remain unclear how well the method would perform in patients with sleep disorders. This limitation was due to the restricted access to abnormal Sleep EEG data with spindle labels.

In terms of future directions, exploring how the SAMC method could be applied to other different types of EEG data, performing various mental tasks rather than spindle detection, would be valuable. Overall, the proposed method better suits those applications with different physiological signals like spikes, sharps, and triphasic waves, among other EEG waveforms [64].

The novel method proposed in this study for spindle detection in sleep EEG signals has great promise. With the ability to map spindle behavior across the scalp, the SAMC method could help unfold the links between spindle types and scalp regions. The parameters used in the spindle identification are malleable to application areas and can be visualized on a heat map, as shown in Figure 3. This implies that experts can see the amplitude of the spindles and the frequency range of the events.

The results presented in this study suggest that the SAMC method can be a helpful tool for sleep researchers and clinicians.

In this study, spindles detection on a single channel was also conducted on all the three DBs. Extra spindles (FP) were often identified using a single-channel method compared to the proposed SAMC method. For some FNs generated by the single channel method, it was found that the duration of those spindle-like waves scored by the expert did not last more than 0.5 s. Some events were fragmented with less than 0.5 seconds in each fragment and separated for more than 0.25 s. It was observed that other FN events had a central frequency outside the spindle frequency range (<11 Hz or >16 Hz).

Furthermore, this study revealed that the performance of our proposed method can be further enhanced when a database contains labels from multiple experts, as evidenced in the Dreams and SS2-MASS DBs. Suggesting that the performance of the proposed method could be improved for the NAP DB if a set of labels were available from an additional score.

### Data Consideration

It is essential to mention that the spindle labels included in NAP, SS2-MASS and Dreams DBs were scored based on the R&K rules [25], which leaves a considerable margin of subjective interpretation, causing label discrepancies between experts [65]. It has been previously documented in [32, 48, 56, 61], and reviewed in this study by comparing expert’s spindles labels from the Dreams and SS2-MASS DB as shown in Table 8.

**Table 8.** Sleep spindle experts’ scoring comparison across databases.

Sleep Spindle Experts’ Scoring Comparison Across Databases:						
Database	Expert 1	Expert 2	Automatic Score (AS)	Agreement	CCC	AR
SS2-MASS DB	9338	15556	---	~9064	0.57	E1: >97% E2: >58%
Nap DB	3516	---	---	---	---	---
Dream DB	298	409	528	Expert1 Vs. Expert2: 159 Experts Vs. AS: 138	Expert1 Vs. Expert2: 0.35 Experts Vs. AS: 0.28	E1:<54% E2:<39% AS:<27%

The average agreement rate between the experts on scoring spindles for the Dreams DB was under 50%. In some cases, like for EEG recordings from Subject 1 and Subject 3, the agreement did not reach 25% and 10%, respectively. And when comparing the agreement rate of the labels between both experts and the automatic method provided by the Dreams DB, the average agreement rate was under 60%.

In the case of the SS2-MASS DB, the average agreement between the two experts was under 58%, with an AR for Expert 1 of 97% and 58% for Expert 2, as documented in Table 8.

## Conclusion

In conclusion, this study presents a new approach for spindle detection in sleep EEG signals that offers promising results. The proposed SAMC method outperforms several existing methods in terms of sensitivity, PPV, and F-score, as demonstrated in our experimental results using three publicly available databases (the NAP, SS2-MASS and Dreams DBs).

The implementation of the SAMC method brings two significant benefits, as it can focus the spindles on a specific frequency range and map their behavior in the scalp through multichannel visualization of spindles. After the model training, this method does not require expert labels or further training as it only relies on the definition of the spindles and related parameters as defined in Table 1. The experimental results for the three different DBs show that the proposed method achieves an overall agreement rate, positive predictive value, F-score, and sensitivity of over 90% for all three DBs, compared to the scores from more than one expert. Furthermore, it is observed that the SAMC method outperformed other existing methods (Kinoshita [62], Patti [21], Tsanas [63] and Devuyst [41]).

The spindles identified by the proposed method can be visualized across all channels (as shown in Figure 3). This is useful for investigating the links and relationships between spindle types and specific brain regions. It helps us have a more accurate and comprehensive understanding of the behaviours of the spindles across the scalp. Our findings indicate that the proposed method can be further improved by including more annotations from an additional expert. It is believed that the SAMC method can significantly advance our understanding of the sleep dynamics of the spindles. It is hoped that this work will inspire more research in this exciting area.

## Data Availability

The datasets used in the current study are open sources and are available in Open Science Framework (OSF) [12], ZENODO [40, 41], and Montreal Archive of Sleep Studies (SS2-MASS) [42].

## Notes

**Ignacio A. Zapata** was born in Bogota Colombia in 1987. He received a bachelor’s degree in information technology from Central Queensland University, Australia, in 2014 and a master’s degree in software engineering and Information Technology from the University Southern Queensland (UniSQ), Australia, in 2018. He is currently a PhD student in the area of Data Science at UniSQ. His research interests lie in Machine Learning, EEG Signal Processing, Data Analysis, and Programming.

**Dr. Yan Li** received her PhD degree from the Flinders University of South Australia, Australia. She is a full professor in the School of Mathematics, Physics and Computing at the University of Southern Queensland, Australia. Her research interests lie in the areas of Artificial Intelligence, Machine Learning, Big Data Technologies, Internet Technologies, Signal/Image Processing etc. Prof Yan Li has published over 240 publications and supervised dozens of PhD completions.

**Peng (Paul)** Wen received his BE, M. E., and PhD degrees in electrical and electronic engineering from Huazhong University of Science and Technology, Wuhan, China, and another PhD degree in biomedical engineering from Flinders University of South Australia, Adelaide, Australia. He is a full professor at the University of Southern Queensland, Australia. His research interests include control systems, biomedical engineering, signal processing, brain modelling, pattern recognition, and machine learning.

**Dr. Evan Jones** has over 20 years of experience in general practice as a medical practitioner, practice owner and integrated health facility developer in Australia. This includes the Health Hub Morayfield, a 15,000 sqm integrated primary care facility and Health Hub Doctors Morayfield which serves over 200,000 current patients on record. He is an Adjunct Associate Professor at the University of the Sunshine Coast with a research interest in neuromodulation. As a Director of the Brain Treatment Centre Australia, Dr. Evan provides clinical oversight of a highly specialized form of tailored TMS which uses AI and machine learning to create individualized treatment protocols to reduce neurological symptoms in adults and children with mental health disorders.

**Shauna Fjaagesund** has over 10 years of experience in government administration and health policy, including commercialization of research within large-scale technology parks. She received her MBA with a specialization in Health Sector Management from the University of the Sunshine Coast, Australia. Ms. Fjaagesund is currently employed as Special Projects Manager within the Health Hub Morayfield, providing research and HDR education support to tenants, including the Brain Treatment Centre Australia and Health Hub Doctors Morayfield. Her research interests include technology integration into primary care health services.

## Implementation Details

The data were pre-processed using the MNE Python Library [43], and the methods were created and implemented in MATLAB. Since there is no record of the duration of the spindles in some databases, we set them to a default duration of 1.5 seconds when comparing the SAMC method results with the experts’ labels.
